# The complete genome of *Delftia tsuruhatensis* BL-MMS-01 bears a novel delftibactin biosynthetic gene cluster

**DOI:** 10.1128/mra.00391-26

**Published:** 2026-06-15

**Authors:** Nathan W. Williams, Paul D. Boudreau

**Affiliations:** 1 Department of BioMolecular Sciences, School of Pharmacy, University of Mississippi 551786, University, Mississippi, USA; University of Southern California, Los Angeles, California, USA

**Keywords:** *Delftia*, delftibactin, biosynthetic gene cluster

## Abstract

*Delftia tsuruhatensis* BL-MMS-01 was isolated from soil collected on the University of Mississippi campus. The genome was assembled as a single circular chromosome. Annotation with the antiSMASH pipeline showed a novel version of the delftibactin biosynthetic gene cluster in the genome, which implies the production of novel delftibactin-like small-molecule siderophores.

## ANNOUNCEMENT

In isolating sphingolipid-producing bacteria using a PCR assay for the serine palmitoyltransferase gene, we stumbled across a *Delftia* strain as a false-positive hit (1). This genus is known for the production of siderophores (2–4), so this strain was genome sequenced to discover any siderophore biosynthetic gene clusters present.

Strain BL-MMS-01 was isolated from soil collected on our campus (34.36521, −89.53331) as previously reported (1). Briefly, 10 µL of 5× diluted PBS/soil suspension was plated on defined medium for siderophores with citric acid agar with streptomycin sulfate (Tokyo Chemical Industry) and nystatin (Alfa Aesar) (5). After incubation at 25°C, colonies appeared and one colony was restreaked onto a fresh plate, affording pure, circular, white-colored colonies. Five milliliters of modified *Acidovorax* Complex Medium (substituting citric acid for pyruvic acid) was inoculated with a colony and incubated at 25°C, 180 rpm shaking, for 48 h (6). A 10% dimethyl sulfoxide (Fisher Bioreagents) stock of the turbid culture was stored at −70°C. Separately, 3 mL of the culture was extracted with the E.Z.N.A. Bacterial DNA kit (Omega Bio-Tek) for V1–V9 16S gene sequencing via a vendor (Azenta’s Bacterial Identification Service). Sanger reads were processed as reported earlier (1), then the 1,344 bp partial 16S gene sequence was deposited with GenBank.

**Fig 1:**
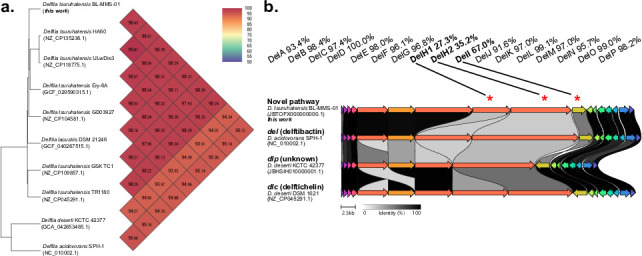
The sequence analysis of the *Delftia tsuruhatensis* BL-MMS-01 genome and delftibactin pathway. (**a**) The OrthoANI heatmap was created with version 0.93.1 of the tool with four threads calculating the orthoANI in both directions with averaging of the result using a genome-to-genome-distance-calculator form of 2 (13). (**b**) The clinker analysis of the delftibactin pathway was carried out on the antiSMASH-annotated (version 8.0.4) pathways on the Comparative Gene Cluster Analysis Toolbox web platform (version 1.0) (14, 15). While protein sequence alignments between the BL-MMS-01 homologs and the *Delftia acidovorans* SPH-1 *del* pathway are indicated above the alignment, these were created by MUSCLE with the PPP algorithm in Geneious (version 2025.2.2). While the genome was similar to other *D. tsuruhatensis* strains, the pathway was distinct from known clusters (2, 4).

A 45 mL culture, grown from the cryogenic stock as above, was extracted with the NucleoBond High Molecular Weight Kit (Macherey-Nagel, DNA Enzymatic Lysis Protocol, adding 10 µL of lysozyme, Omega Bio-Tek); note that no further size selection was used. Nanopore DNA sequencing (R10.4.1 flow cell on a PromethION P24 with the Rapid Barcoding 96 v14 library prep kit) and basecalling (Dorado v4.3, Super-Accurate, with default Q10 quality filtering) by a commercial vendor (Plasmidsaurus, Louisville, KY, USA; Standard Bacteria Genome service) gave 211,493 raw reads with an N50 of 7,763 bp. The raw reads were filtered with Filtlong (v0.2.1), first to a minimum length of 500 bp, keeping the top 99% of reads by quality score, and separately filtering at 1,000 bp/90% (7). The 500 bp/99% reads were assembled via Flye (v2.9.5-b1801) set for nanopore high-quality reads and otherwise the defaults, affording one circular contig (172× mean coverage) (8). This draft was polished with the 1,000 bp/90% reads via medaka (v2.0.1, r1041_e82_400bps_bacterial_methylation configuration, batch size of 32) (9). The polished assembly of 6,277,843 bp, with 66.9% G + C content, was deposited with GenBank. NCBI’s Prokaryotic Genome Annotation Pipeline (v6.10) reported 5,665 genes (5,546 protein coding), 15 rRNA genes, and 80 tRNA genes (10). CheckM2 (v1.1.0) scored the genome as 100% complete with 0.02% contamination (11). The Type Strain Genome Server assigned the BL-MMS-01 to the species *Delftia tsuruhatensis*, the top hit to strain NBRC 16741 (GCA_001571325.1) (12). An OrthoANI Tool-based (v0.93.1) comparison supported that assignment (Fig. 1a) (13). The genome and the comparator *Delftia* were annotated by antiSMASH (v8.0.4) (14), which showed that the siderophore pathway in BL-MMS-01 is unique (Fig. 1b) (2, 4). Changes in the biosynthetic gene cluster suggest this strain produces novel delftibactins.

## Data Availability

The final version of the genome assembly for *Delftia tsuruhatensis* BL-MMS-01, as well as the Sequence Read Archive of the raw vendor reads and preliminary 16S partial gene sequence, is available on GenBank. The accession numbers for these data are JBTOFX000000000 for the genome, SRR36831241 for the Sequence Read Archive, and PX867253 for the 16S sequence.
